# Evaluation of Hyperspectral Indices for Chlorophyll-*a* Concentration Estimation in Tangxun Lake (Wuhan, China)

**DOI:** 10.3390/ijerph7062437

**Published:** 2010-05-27

**Authors:** Yaohuan Huang, Dong Jiang, Dafang Zhuang, Jingying Fu

**Affiliations:** Data Center for Resources and Environmental Sciences, State Key Lab of Resources and Environmental Information System, Institute of Geographical Sciences and Natural Resources Research, Chinese Academy of Sciences, Beijing 100101, China; E-Mails: huangyh@lreis.ac.cn (Y.-H.H.); zhuangdf@igsnrr.ac.cn (D.-F.Z.); fujingying.2005@163.com (J.-Y.F.)

**Keywords:** chlorophyll-*a*, hyperspectral reflectance, hyperspectral indices, Tangxun Lake

## Abstract

Chlorophyll-*a* (Chl-*a*) concentration is a major indicator of water quality which is harmful to human health. A growing number of studies have focused on the derivation of Chl-*a* concentration information from hyperspectral sensor data and the identification of best indices for Chl-*a* monitoring. The objective of this study is to assess the potential of hyperspectral indices to detect Chl-*a* concentrations in Tangxun Lake, which is the second largest lake in Wuhan, Central China. Hyperspectral reflectance and Chl-*a* concentration were measured at ten sample sites in Tangxun Lake. Three types of hyperspectral methods, including single-band reflectance, first derivative of reflectance, and reflectance ratio, were extracted from the spectral profiles of all bands of the hyperspectral sensor. The most appropriate bands for algorithms mentioned above were selected based on the correlation analysis. Evaluation results indicated that two methods, the first derivative of reflectance and reflectance ratio, were highly correlated (R^2^ > 0.8) with the measured Chl-*a* concentrations. Thus, the spatial and temporal variations of Chl-*a* concentration could be conveniently monitored with these hyperspectral methods.

## 1. Introduction

The deterioration of the water quality of inland water bodies has been a serious ecological and social problem in China, since many lakes (both natural and artificial) and rivers are the main sources of drinking water, as well as water for agricultural use. With the increasing shortage of available water resources, the protection and maintenance of water quality have been a primary objective of watershed or water resources management. Eutrophication is a persistent water quality problem affecting the ecological health of many shallow lakes [1]. Lake eutrophication and aquatic ecological degradation have attracted wide attention over the last decades for their harmful influences on human health [2]. Some algae causing eutrophication can produce toxins such as microcystin, which is recognized as an important cause of liver cancer. Besides the toxins in eutrophicated water being harmful for drinking, they can also be accumulated in the body of aquatic foods, such as fish, shrimp, shellfish and so on. Humans and animals who consume contaminated water and food can become poisoned or even worse, killed. Furthermore, if the drinking water sources such as lakes and reservoirs are eutrophicated, the overgrown algae will affect the working of water treatment plants by blocking filters. To remove the smell and toxins of the water and remediate water eutrophication, large amounts of chemicals are often used, which will degrade the quality of tap water and affect drinking water safety, so monitoring eutrophication is important to human health.

Phytoplankton primary production is regard as a reliable and accurate indicator for eutrophication assessment [3]. Numerous researchers, after analyzing the physicochemical and ecological processes of lakes and their effects on phytoplankton [4,5], have found that chlorophyll-*a* (Chl-*a*) is a fundamental proxy for phytoplankton abundance, which is widely used in recent research [6]. Chl-*a* is a green pigment found in plants trapping sunlight for photosynthesis, and its concentration in the aquatic environment is an indicator of phytoplankton abundance, tropic state, and biomass [7].

Chl-*a* has been long applied as a trophic condition index of water bodies. Steele summarized the use of Chl-*a* as an indicator of photoautotrophic biomass based on its relationship with primary productivity [8]. Cullen elaborated the application of Chl-*a* as an index for biomass of primary producers [9]. Chl-*a* biomass reflects the net result of growth and loss in pelagic waters. Algal biomass, which is closely related with planktonic primary production, is a universally acknowledged indicator of trophic state, because it is the visible manifestation and part of the process of eutrophication [10]. Therefore, as the most serious pollution of lakes in China, phytoplankton blooms (or called algal blooms) can be monitored with the index of Chl-*a* [11] and it is accepted that Chl-*a* concentration in a water body is an important index for detecting the degree of pollution in inland water such as lakes, rivers, *etc*. [12].

Although Chl-*a* is relatively easily measured in comparison with algal biomass, Chl-*a* monitoring through *in situ* sampling is costly and time-consuming. The difficulty in achieving continuous water quality sampling is a tremendous barrier in water quality monitoring and forecasting [13]. With the development of remote sensing, especially hyperspectral scanning technology, remote sensing holds significant potential to enhance regional monitoring and assessment of lake water quality and trophic conditions [14], and by offering a useful and cost-effective approach to evaluate Chl-*a*, characterized by rapid results, low costs, and convenience for dynamic monitoring, remote sensing has become an important means to estimate Chl-*a* levels in inland lakes.

Numerous studies have focused on deriving Chl-*a* concentration information from hyperspectral sensor data in inland water bodies. All are based on the properties of the reflectance peak near 700 nm of productive turbid waters. Using vector analysis, Stumpf and Tyler showed that the ratio of the near infra-red (NIR) and the red bands of AVHRR and CZCS can identify phytoplankton blooms and has the potential to provide estimates of Chl-a above 10 mg/m^3^ in turbid estuaries [15]. Gons used the reflectance ratio at 704 and 672 nm, and assessed Chl-*a* concentrations ranging from 3 to 185 mg/m^3^ at these wavelengths [16]. Jiao *et al*. used the reflectance ratio between 719 nm and 667 nm to estimate Chl-*a* concentration in Taihu Lake [17]. Thiemann and Kaufman used the ratio between 705 and 678 nm to assess Chl-*a* in Mecklenburg Lake [18]. Similar algorithms use the ratio between the reflectance peak (R_max_) and the reflectance at 670 nm (R_670_), or the ratio R_705_/R_670_ [19–21]. All of these algorithms used the ratio of the near-infrared (NIR) peak reflectance to the reflectance near 675 nm, which is the red Chl-a absorption band and assumed that optical parameters including Chl-*a* specific absorption coefficient and Chl-*a* fluorescence quantum yield are constant [22,23]. However, the Chl-a fluorescence quantum yield depends on several factors, such as phytoplankton taxonomic composition, illumination conditions, nutritional status, and so on. This makes the bands chosen to estimate Chl-a by the algorithms mentioned above vary in different study areas. The uncertainty of modeling bands is the biggest problem of practical application of the algorithms. Some other semi-analytical algorithms were developed to estimate chlorophyll concentration in turbid waters. Dall’ Olmo revised a three-band reflectance model, originally developed for pigment content estimation in terrestrial vegetation, to assess Chl-*a* in turbid productive waters [22,24,25]. Le extended the model to four-bands and applied it to estimate Chl-*a* in Taihu Lake [26]. Although the influential factors of the algorithm are analyzed, it is also difficult to choose optimal band positions for Chl-*a* estimation in different lakes, because the spectrum of lakes is varying with different bandwidths, lakes and seasons.

For all the limitations mentioned above, identification of the best indices for Chl-*a* monitoring is a current research issue. The objective of this study was to assess the potential of hyperspectral indices for Chl-*a* concentration detection in Tangxun Lake. The specific goals were: (1) to select the most appropriate bands for calculating several types of hyperspectral indices; (2) to evaluate different types of models in terms of their sensitivity to Chl-*a* concentration.

## 2. Study Area and Measurements

### 2.1. Study Area

In this study, Tangxun Lake was selected as the study area. Located between 30°22′N and 30°30′N and between 114°15′E and 114°35′E, in Wuhan, Central China, Tangxun Lake, as the second largest lake in Wuhan City, has a storage capacity of 32.85 million km^3^ and a catchment area of 240.38 km^2^. Its current surface area is approximately 32.85 km^2^, accounting for 28.7% of the total lake area in Wuhan. Tangxun Lake is a typical shallow lake with a mean water depth of 18.5 m. With the industrialization and urbanization of Wuhan, an enormous amount of untreated wastewater and sewage was discharged into the lake without treatment, resulting in an incessant increase of nutrient concentration in the lake. Consequently, water eutrophication has become a serious environmental problem, and the water quality of Tangxun Lake, as a result, can barely satisfy the ecological and living requirements and it is thus necessary to develop methods for monitoring the Chl-*a* concentration both temporally and spatially.

### 2.2. In-situ Measurements

**Figure 1.**
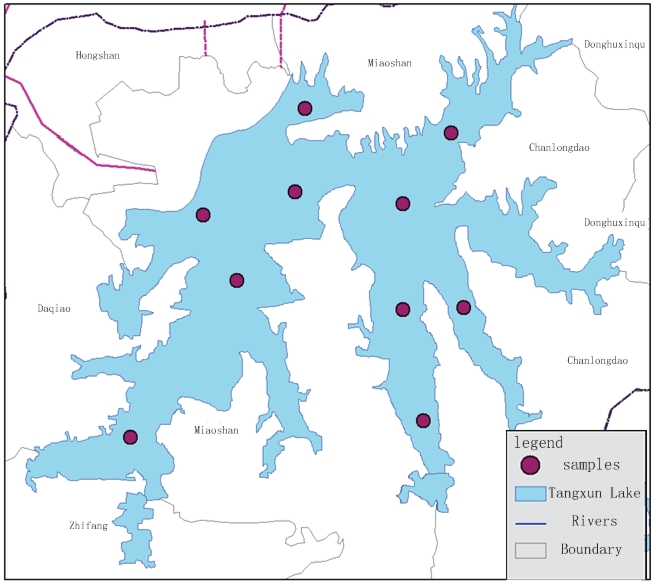
Study area and sampling locations.

Two datasets of hyperspectral reflectance data and Chl-*a* concentration data were used in this study. The field work was carried out between 10:00 and 12:00 h local time on April 16, 2009. The two types of data were collected simultaneously. The location of Tangxun Lake and that of the ten sampling sites are shown in Figure 1.

**Figure 2.**
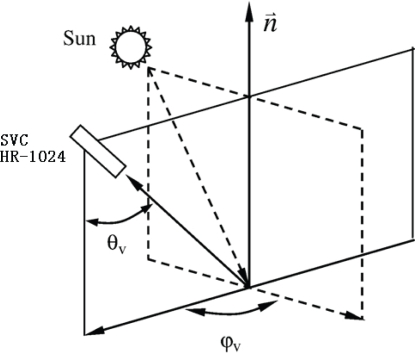
Viewing geometry of spectra sampling.

Hyperspectral reflectance was measured with a SVC HR-1024 spectroradiometer with a spectral resolution of less than 3.5 nm in the spectral range from 350 to 1,000 nm. The spectral resolution was resampled by the equipment’s software. In the measurement, the instrument was held manually over the deck of an anchored ship approximately one meter above the water surface. At each sampling location, three kinds of radiances were measured: radiances of water surface (*L_sw_*), a standard grey board (*L_p_*), and skylight (*L_sky_*). As shown in Figure 2, the instrument was positioned at an angle *ϕ_v_* of 90–135° with the plane of the incident radiation away from the sun. The view of the water surface *θ_v_* was controlled between 30 and 45° with the aplomb direction. Immediately after measuring water radiance, the spectroradiometer was rotated upwards by 90–120° to measure *L_sky_*. The view zenith angle in this measurement was kept the same as that in measuring water radiance. Contrary to the other two radiances, *L_sw_* was measured ten times at each sampling location. Reflectance spectra were checked and the dataset with obvious errors were abandoned. Then the average of the remaining dataset was used as the value of field measurements. Hyperspectral reflectance is defined as:

(1) $$\{\begin{array}{l} R_{rs}=\frac{L_{w}}{E_{d}(0^{+})}\\ L_{w}=Lsw-rL_{sky}\\ E_{d}(0^{+})=\pi L_{\text{p}}/\rho_{\text{p}} \end{array}$$

where *L_w_* is the water-leaving radiance; *E_d_* (0^+^) is the total incident radiance flux of water surface; *L_sw_* stands for total radiance received from the water surface; *L_sky_* refers to diffused radiation of the sky; *r* represents the reflectance of skylight on the air water interface, depending upon solar azimuth, measurement geometry, wind speed, and surface roughness; *L_p_* is the radiance of the gray board; and *ρ_p_* stands for the reflectance of the gray board [26].

Surface water samples (2.5 L volume) were collected at a depth of 0.5 m below the surface immediately after the reflectance measurements. These samples were stored in a cooler with ice in the dark, and taken back to the laboratory for Chl-*a* concentration analysis within 6 hours. The pigment samples were extracted in hot (80 °C) 90% ethanol, and Chla concentration was quantified fluorimetrically.

## 3. Analysis

### 3.1. Reflectance Spectra Analysis

**Figure 3.**
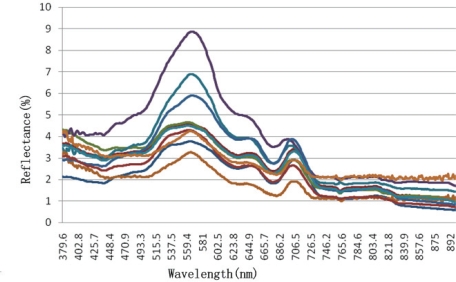
Reflectance spectra at 10 sampling sites in Tangxun Lake.

We selected the spectroradiometer data with wavelengths between 380 and 900 nm for analysis according to previous research. The spectral reflectance curves of the samples are shown in Figure 3.

The reflectance spectra show a clear reflectance peak around 570 nm, which then decreases gradually. Another peak appears around 706 nm. The curves show less diversity when the wavelength is larger than 730 nm. According to previous studies, the reflectance peak near 570 nm may be caused by low absorption of algal pigments or the scattering of inorganic suspended materials and phytoplankton cells. The absorption valley from 670 nm to 686 nm may be caused by the maximum absorption of chlorophyll-a in the red-band. The other reflectance peak near 706 nm may be due to fluorescence of Chl-*a* [20,27–29]. The magnitude and shape of the reflectance curves (Figure 3) are all similar to that of typical turbid water.

### 3.2. Correlation Analysis

Three types of hyperspectral indices, including single-band reflectance, first derivative of reflectance and reflectance ratio, were extracted from spectral profiles involving all bands of hyperspectral sensor. Correlation analysis was conducted to identify the most appropriate bands for those algorithms.

#### 3.2.1. Single band reflectance VS Chl-*a* concentration

**Figure 4.**
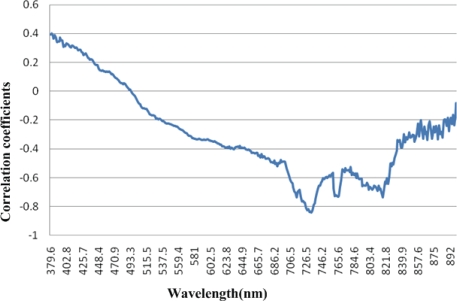
The curve of correlation coefficients between reflectance and Chl-*a* concentration.

The correlation analysis was conducted between Chl-*a* concentration and reflectance of all 384 bands from 380 nm to 900 nm. The curve of correlation coefficients of 384 bands is shown in Figure 4.

It is shown in Figure 3 that reflectance between 380 and 500 nm is positively correlated with Chl-*a* concentration. A negative correlation occurs when the wavelength is larger than 500 nm. The largest negative coefficient appears between 726.5 nm and 734.4 nm (Table 1).

All seven correlation coefficients between reflectance and Chl-*a* concentration between 726.5 nm and 734.4 nm are larger than 0.8. For Chl-*a* estimation based on remote sensing in Tangxun Lake, the optimal bands of the single-band model are near-infrared and the most relevant band is around 733.1 nm.

#### 3.2.2. First-derivative of reflectance VS Chl-*a* concentration

The first-derivative of reflectance can be calculated as [30–33]:

(2) $$R{\left(\lambda_{i}\right)}^{\prime }=\frac{R(\lambda_{i+1})-R(\lambda_{i-1})}{\lambda_{i+1}-\lambda_{i-1}}$$

**Figure 5.**
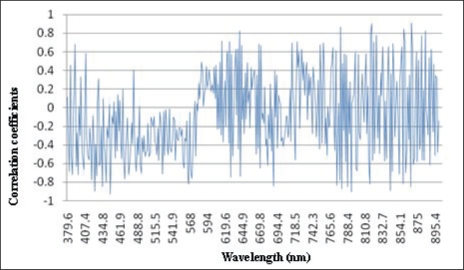
The correlation coefficients between first-derivative of reflectance and Chl-*a*.

In the formula, *R(λ_2_)′* is first-derivative of reflectance in band *λ_2_*; *R*(*λ_2+1_)* and *R*(*λ_2−1_)* are the reflectance of band *λ_2+1_* and band *λ_2−1_*, respectively; and *λ_2+1_* and *λ_2−1_* are the band wavelengths. Correlation analysis was conducted between first-derivative of reflectance and Chl-*a* concentration with Formula 2, and the results are shown in Figure 5.

It is shown in Figure 4 that various bands with higher absolute correlation coefficients exist between first-derivative reflectance and Chl-*a* concentration. The number of correlation coefficients greater than 0.8 is more than that of the single-band analysis result. The strongest correlation appears for the bands at 446.9 nm, 793.4 nm, 819.4 nm and 868.1 nm, with correlation coefficients of −0.929, −0.902, 0.902, and 0.906, respectively, indicating that the optimal bands for Chl-*a* concentration estimation by first-derivative algorithm are adjacent to the blue and NIR region. The absolute value of the correlation coefficient for the band near 446.9 nm is the largest, consistent with the conclusions concerning the absorption valley caused by Chl-*a* absorption [34].

#### 3.2.3. Reflectance ratio VS Chl-a concentration

**Figure 6.**
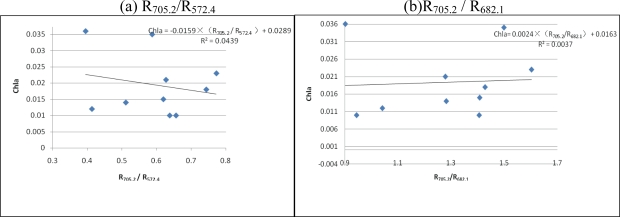
Linear models with reflectance ratios of characteristic bands.

The reflectance ratio of characteristic bands is widely used to estimate Chl-*a* concentration. The reflectance ratio of the peak and valley spectra near 700 nm or 570 nm was applied in various studies. To validate the feasibility of the algorithm on the reflectance ratio of characteristic bands for Chl-*a* concentration estimation in Tangxun Lake, two combinations of R_705.2_/R_682.1_ and R_705.2_/R_572.4_ were applied in linear modeling. The results of linear regression models with the two combinations are shown in Figure 6.

**Figure 7.**
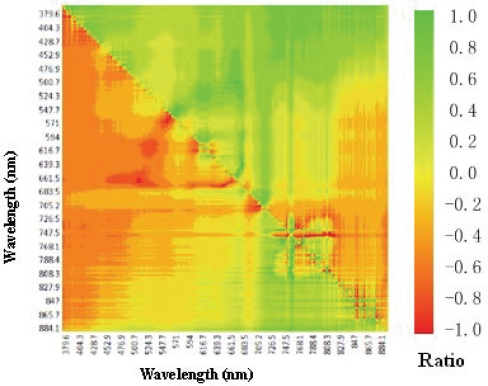
The correlation coefficients between Chl-*a* and reflectance ratios.

As shown in Figure 6 the Chl-*a* concentration estimation does not produce good results on reflectance ratios of characteristic bands, with R^2^ of 0.0439 and 0.0037 respectively in Tangxun Lake. The fitting line will cause a huge error in Chl-*a* concentration estimation, which could not provide the accuracy needed for practical Chl-*a* concentration monitoring. Therefore, it is concluded that reflectance ratios of characteristic bands are not universally applicable. To find the optimal bands suitable for the method for estimating reflectance ratio in Chl-*a* concentration in Tangxun Lake, correlation analysis between the reflectance ratios of different bands and Chl-*a* concentration in field measurements was applied. Reflectance ratios between 384 pairs of bands from 380 nm to 900 nm were calculated for correlation analysis. The distribution of correlation coefficients between reflectance ratios and Chl-*a* are shown in Figure 7. The absolute values of correlation coefficients varied from 0 to 0.928 with different band combinations, and the maximum absolute value of 0.928 was achieved for the R_861.1_/R_865.7_ ratio combination. Based on the correlation analysis, the optimal band combination is easy to obtain, and can be used for Chl-*a* concentration estimation in Tangxun Lake.

According to the correlation analysis results, the R_861.1_/R_865.7_ reflectance ratio with the maximum correlation coefficient of Chl-*a* concentration was selected for linear modeling. For the measured data, the R^2^ of the model reached 0.8605. The model based on correlation analysis was better than the one with empirical characteristic band combinations. The reflectance radio of different bands reduces the noise for Chl-*a* concentration estimation and improves the reflectance information of Chl-*a*, which can be used to improve the accuracy of Chl-*a* retrieval. Reflectance ratio model shown in Figure 6 is different from the models established in our studies. It indicates that the optimal bands should be indentified before application of reflectance radios for Chl-*a* estimation.

## 4. Results

The objective of this study is to obtain the optimal model and its most suitable band combination for Chl-*a* concentration estimation based on remote sensing data in Tangxun Lake. Linear regression models were adopted in the paper for comparison. As shown in Table 2, distinct bands are selected with accuracy for three models.

The bands between 726.5 nm and 734.4 nm are selected for Chl-*a* concentration in Tangxun Lake with the single-band algorithm, and the R^2^ of the linear model is approximately 0.705. For the first-derivative algorithm, the optimal band for Chl-*a* retrieval is at 446.9 nm with a highest R^2^ value of 0.863. And R_861.1_/R_865.7_ ratio is the best band combination for the reflectance radio algorithm, whose accuracy is slightly lower than first-derivative one with a R^2^ value of 0.861. The mean relative error (MRE) is also calculated for three kinds of models. The MRE is expressed as:

(3) $$MRE=\frac{1}{n}\sum_{i}^{n}\frac{\left|y_{i}-f(x_{i})\right|}{y_{i}}*100\%$$

where *y_i_* is the ith observed test sample value, *n* is the number of samples and *f(x_i_)* is the estimated ith value.

**Figure 8.**
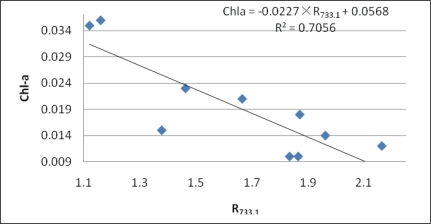
Scatter plots of Chl-*a versus* reflectance at 733.1 nm.

**Figure 9.**
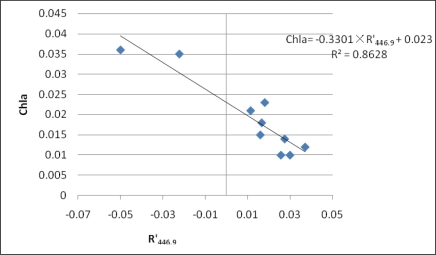
Scatter plots of Chl-*a versus* first-derivative of reflectance at 446.9 nm.

**Figure 10.**
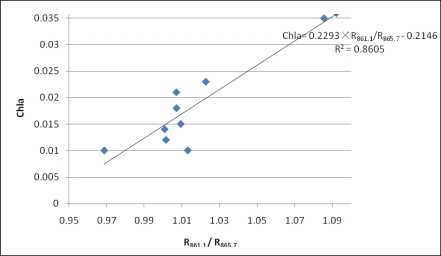
Scatter plots of Chl-*a versus* reflectance ratio R_861.1_/R_865.7_.

The MRE of first-derivative model is the lowest with a value of 11.2%, and the MRE of reflectance ratio model is slightly higher, with a value of 13.8%. Meanwhile, the largest error exists in the single-band model with an MRE of 26.3%. It can therefore be concluded that it is feasible to to estimate Chl-*a* concentrations in Tangxun Lake with the algorithms of first-derivative and reflectance radio, which are better than the single-band model. While in practical Chl-*a* monitoring, it is necessary to select the most suitable one according to the available data. The results of three kinds of regression models are shown in Figure 8, Figure 9 and Figure 10 respectively.

## 5. Conclusions

In this study, the potential of hyperspectral data to derive Chl-*a* concentration in inland waters is discussed. Based on the field measurements in Tangxun Lake, Central China, it is found that three characteristic points of the reflectance spectra exist with reflectance peaks adjacent to 570 nm and 706 nm and an absorption valley ranges from 671 nm to 686 nm. The wavelengths of the characteristic bands are slightly different from those reported in previous studies, which may be attributed to differences in the study area and season.

The correlation analysis has indentified the proper wavelengths or band ratios for the extraction of Chl-*a* concentrations in water. The algorithms of single-band, first-derivative, and reflectance radio were applied and evaluated. Although the single-band algorithm is simple, it is hard to estimate Chl-a concentrations comprehensively. The reflectance radio algorithm for estimating Chl-a concentrations is well-documented because it can highlight the spectral characteristics of absorption and reflection and then reduce the effect of noise. The R_NIR_/R_RED_ radio was commonly used in previous research, and, for example, Mittenzwey shown a high coefficient of determination (R^2^) of 0.98 between chlorophyll and the near-infrared (NIR)/red reflectance ratio [35], but in our study it was shown that the results of using R_NIR_/R_RED_ were poor, with an R^2^ value of 0.043, which indicated the _NIR_/R_RED_ is not suitable to estimate Chl-a concentrations in Tangxun Lake. This agrees with the conclusions of Han’s study in Branched Oak Lake [31]. In our case, the reflectance ratio between 861.1 nm and 856.7 nm were correlated significantly with Chl-a concentration (R^2^ > 0.86). Derivative spectra indicate the rate of change of reflectance with wavelength, so derivative analysis allows one to correlate the shape of the reflectance pattern to Chl-a concentrations. The first-derivative algorithm can reduce pure-water effects on the water effect [36] and is an objective tool for isolating the absorption features of phytoplankton [37]. It had been applied by researchers in estimating Chl-a concentrations in water. The first-derivative at near 690 nm is found to be useful in modeling Chl-a concentration [31]. We found the first-derivative of reflectance at 446.9 nm was most appropriate to estimate Chl-a concentrations in Tangxun Lake. This agrees with Fraser’s finding that the first-derivatives near 440 nm was also correlated significantly with Chl-a [38]. The first-derivative model was the best in estimating Chl-a concentrations in Tangxun Lake with R^2^ and MRE of 0.863 and 11.2%, respectively. The difference of the most appropriate bands in our study means that it is of primary importance to choose optimal bands for estimating Chl-a concentrations in specific waters. The results suggest that assessment of Chl-*a* concentration using hyperspectral indices has significant potential. In recent years, more and more hyperspectral sensors data are becoming available. The method presented in this study will help enable departments concerned to continuously monitor water quality with high accuracy in large areas.

## Figures and Tables

**Table 1.** The correlation between spectral reflectance and Chl-*a* concentration from 726.5–734.4 nm.

**Wavelength (nm)**	726.5	727.8	729.1	730.5	731.8	733.1	734.4
**Correlation coefficients**	−0.80652	−0.82203	−0.815	−0.83498	−0.83097	−0.83998	−0.82338

**Table 2.** Regression models for Chl-*a* with three types of spectrum indices.

**Model**	**Optimal Bands**	**Model**	**R^2^**	**MRE**
Single-band	733.1 nm	Chla = −0.0227×R_733.1_ + 0.0568	0.705	26.3%
First-derivative	446.9nm	Chla = −0.3301R’_446.9_ + 0.023	0.863	11.2%
Reflectance Ratio	R_861.1_/R_865.7_	Chla = 0.2293 × (R_861.1_ / R_865.7_) − 0.2146	0.861	13.8%
